# The Potential Role of Probiotics in Controlling Overweight/Obesity and Associated Metabolic Parameters in Adults: A Systematic Review and Meta-Analysis

**DOI:** 10.1155/2019/3862971

**Published:** 2019-04-15

**Authors:** Zhi-Bin Wang, Shan-Shan Xin, Li-Na Ding, Wen-Yu Ding, Yan-Li Hou, Chang-Qing Liu, Xian-Dang Zhang

**Affiliations:** ^1^Shandong Institute of Endocrine and Metabolic Diseases, Shandong Academy of Medical Sciences, Jinan, Shandong, China; ^2^Jinan Center for Food and Drug Control, Jinan, Shandong, China

## Abstract

**Background:**

The prevalence of overweight/obesity in adults is raised to 39%, which is nearly tripled more than 1975. The alteration of the gut microbiome has been widely accepted as one of the main causal factors. To find an effective strategy for the prevention and treatment of overweight/obesity, a systematic review and meta-analysis were designed.

**Methods:**

In this study, we systematically reviewed the article published from January 2008 to July 2018 and conducted a meta-analysis to examine the effects of probiotics on body weight control, lipid profile, and glycemic control in healthy adults with overweight or obesity. The primary outcomes were body weight, body mass index (BMI), waist circumference, fat mass, fat percentages, plasma lipid profiles, and glucose metabolic parameters.

**Results:**

We systematically searched PubMed, Embase, and the Web of Science and identified 1248 articles, and 7 articles which were manually searched by the references of included studies and previously systematic reviews. Twelve randomized controlled trials (RCTs), including 821 participants, were included in the meta-analysis via full-text screening. Probiotics supplementation resulted in a statistical reduction in body weight (WMD [95%* CI*]; -0.55 [-0.91, -0.19] kg), BMI (WMD [95%* CI*]; -0.30 [-0.43, -0.18] kg m^−2^), waist circumference (WMD [95%* CI*]; -1.20 [-2.21, -0.19] cm), fat mass (WMD [95%* CI*]; -0.91 [-1.19, -0.63] kg), and fat percentage (WMD [95%* CI*]; -0.92 [-1.27, -0.56] %) compared with control groups. As expected, the metabolic parameters were improved significantly, with a pooled standardized mean difference in TC (SMD [95%* CI*]; -0.43 [-0.80, -0.07]), LDL-C (SMD [95%* CI*]; -0.41 [-0.77, -0.04]), FPG (SMD [95%* CI*]; -0.35 [-0.67, -0.02]), insulin (SMD [95%* CI*]; -0.44 [-0.84, -0.03]), and HOMA-IR (SMD [95%* CI*]; -0.51 [-0.96, -0.05]), respectively. The changes in TG (SMD [95%* CI*]; 0.14 [-0.23, 0.50]), HDL-C (SMD [95%* CI*]; -0.31 [-0.70, 0.07]), and HbA1c (SMD [95%* CI*]; -0.23 [-0.46, 0.01]) were not significant.

**Conclusion:**

This study suggests that the probiotics supplementation could potentially reduce the weight gain and improve some of the associated metabolic parameters, which may become an effective strategy for the prevention and treatment of obesity in adult individuals.

## 1. Introduction

Overweight/obesity is one of the most widespread chronic diseases around the world with the character of excessive energy intake and insufficient energy expenditure [1, 2] and with an elevated risk of several chronic diseases, including type 2 diabetes, hyperlipidemia, hypertension, and cancer [3–5]. Recent studies revealed that the occurrence of obesity is associated with the gut microbial dysbiosis, which might induce the alteration of the host's energy absorption and influence intestinal permeability, and the fasting-induced adipose factor [3, 4, 6, 7].

Interestingly, emerging evidence suggests that the probiotics are the organic component of the gut microflora and could meliorate the gut microbiota [3, 4]. As a kind of active microorganism, the probiotics regulate the intestinal microecosystem, improve the gut microecosystem and the host energy metabolism, and reduce the chronic inflammation and oxidative stress [4, 8, 9]. These studies indicate that probiotics may play a role in the prevention and treatment of obesity by regulating the gut microbiota [4, 10, 11]. Overweight/obesity, which is associated with the loss of glycemic control and dyslipidemia, could seriously affect the patients' life quality and increase their economic burden [1, 9, 12]. However, the effect of probiotics to control the body weight and related clinical indicators in healthy adults with obesity are remaining unclear. Several randomized controlled studies evaluated the effects of probiotics on body weight control, lipid profiles, and glycemic control and gave conflicting results; several studies suggest that probiotics play an important role in the prevention of obesity [13–19], while other studies hold different views [20–22]. Studies indicated that overweight/obesity is usually associated with elevated levels of plasma lipid profiles concomitant with impaired glucose metabolism [23, 24]. However, no previous review has assessed the effect of probiotics on plasma lipid profiles and glycemic parameters. According to previous studies, multiple factors could influence the effects of probiotics [23, 25, 26]. To evaluate the obesity controlling effect of probiotics, we conducted the systematic review and meta-analysis on the correlation of probiotics and plasma lipid profiles, glucose metabolic parameters.

## 2. Methods

### 2.1. Search Strategy

Two reviewers independently executed a systematic literature search on 16 July 2018 from the databases of PubMed, Embase, and Web of Science. The search was limited to the clinical randomized controlled trials (RCTs). For search strategies designed for PubMed, Embase, and Web of Science, we used the controlled vocabulary terms for each concept (e.g., MeSH) and keyword synonyms (see Table S1 for exact search strategies). We also manually searched the references of included studies and previously systematic reviews to identify further relevant studies.

### 2.2. Eligibility Criteria

Studies were selected on the basis of the following criteria: (1) overweight and obesity were defined by body mass index (BMI); only the studies with individuals with a BMI > 25 kg m^−2^ were included; (2) only the studies with general healthy individuals were included; (3) randomized controlled trials (RCTs) of adults (≥18 years old) were included; (4) changes in weight-loss in adults between pre- and postintervention by probiotics consumption were the primary outcomes, and the lipid profiles and glucose metabolic parameters were considered as the secondary outcomes. In the event when there were multiple intervention groups (multiple strains or multiple dosages of probiotics) in one study, only the largest number of strains or dosage of probiotics group and placebo group were included [27].

The exclusion criteria were (1) studies with breast-breeding or pregnant patients; (2) patients with diabetes, hypertension, chronic immunologic diseases, thyroid diseases, gastrointestinal surgery, or other chronic disease; (3) the trials with participants who consumed prebiotics, synbiotics, herb, and other supplements (such as micronutrients or other dietary constituents).

### 2.3. Data Extraction

Two investigators independently executed the literature search, data extraction, and quality assessment based on the eligibility criteria. Any disagreements were resolved by discussion between data collectors alone with a third investigator. Data on year, country, study design, population, types of probiotics administration, duration of treatment, and clinical outcomes were extracted from the included studies.

### 2.4. Risk of Bias with Individual Studies

The risk of bias within randomized controlled trials was independently evaluated by two investigators. The risk of selection bias, performance bias, detection bias, attrition bias, reporting bias, and other biases of the included trials was assessed as high, low, or unclear, using the Cochrane Collaboration tool [28].

### 2.5. Statistical Analysis

The differences in the mean change from baseline in body weight/BMI/waist circumference/fat mass/fat percentage/glycaemic factors/lipid profiles comprised the primary measure of treatment effect. The meta-analysis was conducted via Review Manager 5.3. It was a significant result when the* P* value was less than 0.05. The changes between baseline and after intervention on weight-loss (e.g., body weight, BMI, waist circumference, fat mass, and fat percentage) were analyzed by the weighted mean difference (WMD). While the changes in lipid and glucose metabolic parameters were analyzed by the standardized mean difference (SMD), the results of lipid and glucose metabolic parameters were measured in a variety of ways [29]. The mean change (standard deviation) in weight-loss and lipid and glucose metabolic parameters from baseline was used to calculate the mean difference (95% confidence interval [CI]) between intervention groups and control groups. When not provided by the authors, we calculated the standard deviation (SD) of the mean change using the correlation coefficient formula in the succeeding text [29]. Imputation of the SD includes three steps: (1) calculate the correlation coefficient (Corr) between the baseline and final values for each intervention group and control group from the included trials; (2) take the average of these Corrs as the imputed Corr; (3) impute the SD of mean change with the imputed Corr [29].

(1) SDChange=SD2Baseline+SD2Final−2×Corr×SDBaseline×SDFinal

Results from all the RCTs were used to calculate the WMD or SMD using a random effects model. Heterogeneity among the included studies was evaluated by Cochrane Q-test and I square (*I*^*2*^).* I*^*2*^ values of 25%, 50%, and 75% were considered as low, moderate, and high level of heterogeneity [30]. Subgroup analyses and sensitivity analyses were conducted based on probiotics dosage, numbers of probiotics species, and forms of probiotics, except that the included studies were less than 7. Sensitivity analyses were also conducted by omitting one trial at a time from the included studies, thereby assessing its effect on the WMD or SMD. The funnel plot and Egger's regression test were conducted by STATA/IC 15.0 to assess the possible publication bias of the included studies. There was no publication bias if the *P* value was more than 0.1 in Egger's regression test.

## 3. Results

### 3.1. Search Results and the General Characteristics of Included Studies

**Figure 1:**
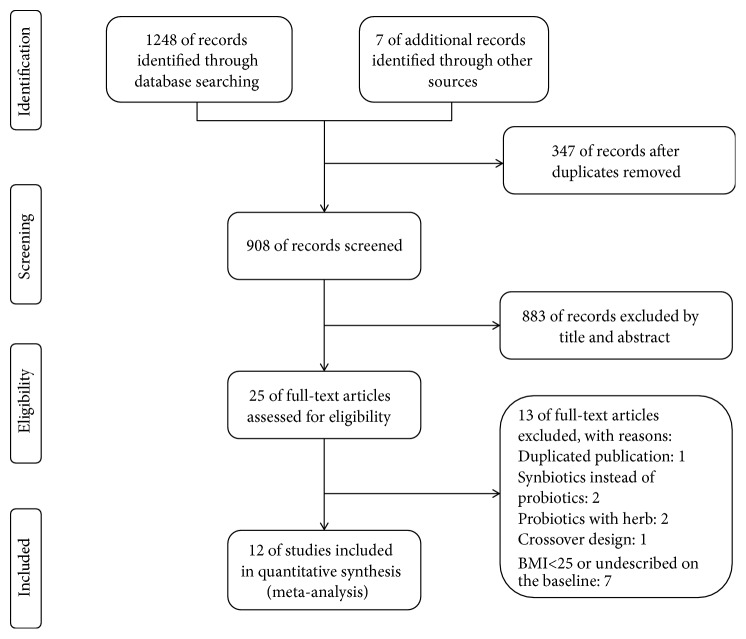
Study flow diagram for study selection.

A total of 1255 literatures (191 from PubMed, 719 from Embase, 338 from Web of Science, and 7 from references) were identified using the search strategy previously described in the method part, from which 347 were excluded after duplicate deletion (Figure 1). Two investigators independently identified 25 articles by screening the title and abstract using the eligibility criteria as described previously. Of the 25 studies, 13 studies did not match the eligibility criteria; 12 RCTs including 11 randomized, double-blinded, controlled trials and 1 randomized, single-blinded, controlled trial were included in the meta-analysis.

The general characteristics of the 12 included studies are presented in Table 1, of which 7 studies included participants who consumed two or multiple strains of probiotics, and 5 studies included participants who consumed a single strain of probiotics. 7 studies investigated a high dosage of probiotics (>10^10^ CFU) and 5 studies investigated lower dosage of probiotics (<10^10^ CFU). Among the 12 included studies that comprised a total of 821 subjects, 416 participants were given placebo and 405 participants were given probiotics. Probiotics were administered in different forms, including sachet, capsule, powder, kefir, yogurt, and fermented milk. Duration of the probiotics supplementation ranged from 8 to 24 weeks.

### 3.2. Risk of Bias with Individual Studies

**Figure 2:**
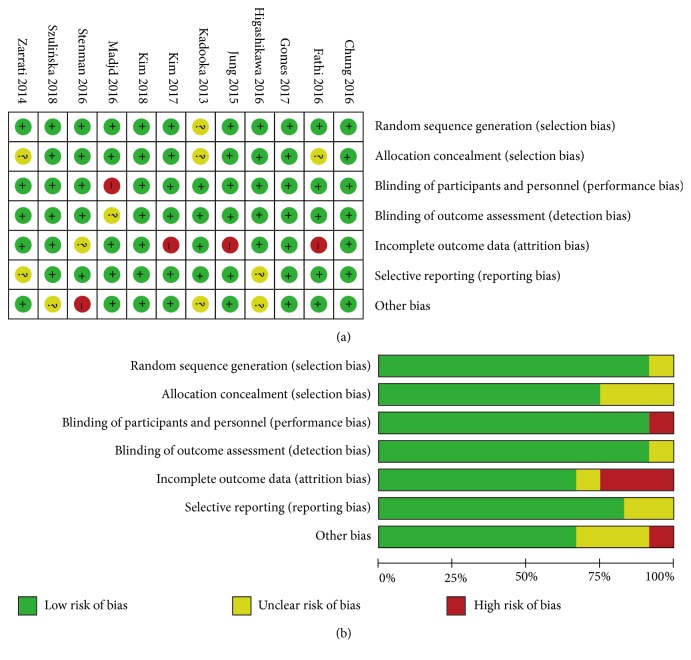
Risk of bias assessment: (a) details of included studies; (b) overall summary.

The Cochrane Collaboration tool for assessment of randomized controlled trials was independently carried out to assess the risk of bias among the included studies by two authors. There was no significant selection bias, performance bias, detection bias, attrition bias, reporting bias, or other biases detected among the included trials (Figure 2).

### 3.3. Effects of Probiotics on Weight-Loss and Associated Metabolic Parameters

#### 3.3.1. Probiotics and Weight-Loss (Body Weight, BMI, Waist Circumference, Fat Mass, and Fat Percentage)

**Figure 3:**
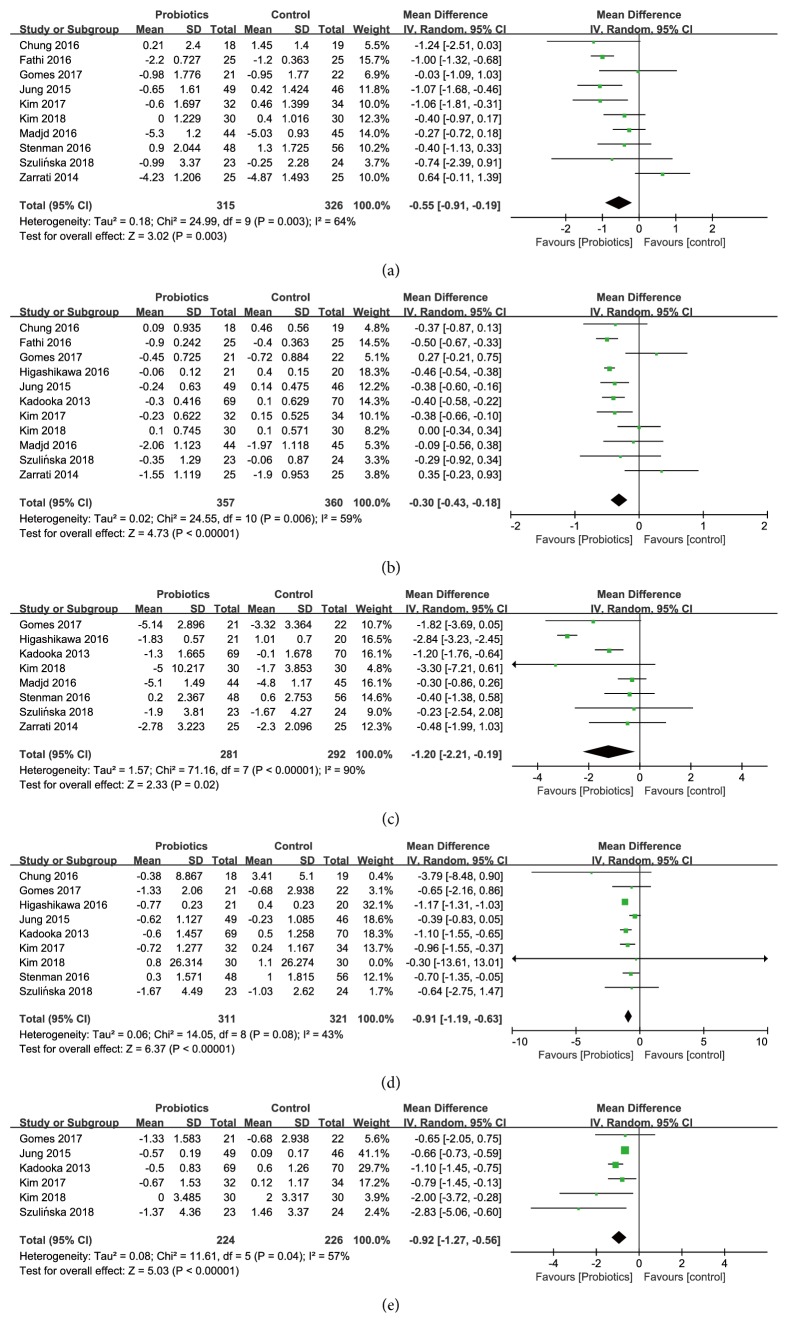
Forest plot of effect of probiotics on (a) body weight; (b) BMI; (c) waist circumference; (d) fat mass; (e) fat percentage.

*Effects on Body Weight*. The overall estimates of the 10 studies among 641 participants (315 consuming probiotics, 326 not consuming probiotics) with changes in body weight showed a statistically significant body weight reduction (WMD [95%* CI*]; -0.55 [-0.91, -0.19] kg,* P* = 0.003) in the probiotics group, comparing with the control group (Figure 3(a)), and there was a moderate heterogeneity (*I*^*2*^ = 64%,* P* =0.003). Subgroup analyses (Table 2) stratified by probiotics dosage, the number of probiotics strains, or forms of probiotics showed the effects of probiotics supplementation on body weight were significantly reduced in trials with high dose of probiotics (-0.58 [-0.92, -0.23] kg), a single strain of probiotics (-0.49 [-0.92, -0.07] kg), and the capsule or powder of probiotics (-0.55 [-0.84, -0.26] kg). Sensitivity analyses revealed that no particular studies significantly affected the summary effects of body weight.

There was no significant publication bias analyzed by Egger's test for the effect of probiotics on body weight (*P* = 0.446), and the funnel plot was presented in Figure S1.

*Effects on Body Mass Index (BMI)*. 11 studies, among 717 participants (357 consuming probiotics, 360 not consuming probiotics), reported the effect of probiotics supplementation on BMI (Figure 3(b)). Comparing with the control group, the reduction of BMI was statistically significant with a pooled weighted mean difference of -0.30 kg m^−2^ (WMD [95%* CI*]; [-0.43, -0.18],* P* < 0.00001) in the intervention group with a moderate heterogeneity (*I*^*2*^ = 59%,* P* = 0.006) between the studies.

Subgroup analyses (Table 3) stratified by probiotics dosage, the number of probiotics strains, or forms of probiotics showed the effects of probiotics supplementation on BMI were significantly reduced with the high dose (-0.29 [-0.46, -0.12] kg m^−2^) and single strain of probiotics (-0.36 [-0.52, -0.20] kg m^−2^). However, the reduction was not significantly associated with two subgroups stratified by the forms of probiotics. Sensitivity analyses revealed that no particular studies significantly affected the summary effects of BMI.

The risk of publication bias analyzed by Egger's test for the effect of probiotics on BMI (Egger's test* P*=0.006) was high, and the funnel plot was presented in Figure S1. To identify and correct the publication bias, five hypothetical negative unpublished trials were conservatively imputed to mirror the positive trials that caused funnel plot asymmetry via the “trim and fill” method. The reduction of BMI incorporating the hypothetical trials continued to be statistically significant with a pooled weighted mean difference of -0.44 kg m^−2^ (WMD [95%* CI*]; [-0.57, -0.30],* P* < 0.0001) in the intervention group. The funnel plot produced by imputed trials was presented in Figure S1. 

*Effects on Waist Circumference*. Eight studies, among 573 participants (281 consuming probiotics, 292 not consuming probiotics), reported the effect of probiotics supplementation on waist circumference (Figure 3(c)). The reduction of waist circumference was statistically significant with a pooled weighted mean difference of -1.20 cm (WMD [95%* CI*]; [-2.21, -0.19],* P* = 0.02) in the intervention group compared with the control group, with a high level of heterogeneity (*I*^*2*^ = 90%,* P* < 0.00001) between the studies. Subgroup analyses (Table 4) stratified by probiotics dosage, the number of probiotics strains, or forms of probiotics indicated the effects of probiotics supplementation on waist circumference were significantly reduced in trials with high dose of probiotics (-1.53 [-2.64, -0.41] cm), a single strain of probiotics (-1.69 [-3.04, -0.33] cm), and the food form of probiotics (-1.11 [-1.64, -0.59] cm). Sensitivity analyses revealed that no particular studies significantly affected the summary effects of waist circumference.

There was no sign of publication bias detected by Egger's test for the effect of probiotics on waist circumference (*P* = 0.403), and the funnel plot was presented in Figure S1. 

*Effects on Fat Mass and Fat Percentage*. A total of 9 studies including 632 participants (311 consuming probiotics, 321 not consuming probiotics) evaluated the effect of probiotics supplementation on fat mass (Figure 3(d)), and 6 studies with 450 participants (224 consuming probiotics, 226 not consuming probiotics) reported changes in fat percentage (Figure 3(e)). Probiotics significantly reduced the fat mass (WMD [95%* CI*]; -0.91 [-1.19, -0.63] kg,* P* < 0.00001) in the intervention group with a moderate level of heterogeneity (*I*^*2*^ = 43%,* P* = 0.08). A pooled effect of fat percentage in the intervention group was also significant (WMD [95%* CI*]; -0.92 [-1.27, -0.56] %,* P* < 0.00001) with a moderate level of heterogeneity (*I*^*2*^ =57%,* P *=0.04).

Subgroup analyses stratified by probiotics dosage, the number of probiotics strains, and forms of probiotics indicated that the effect of probiotics supplementation on fat mass was significantly reduced (Table 5), showing a greater decrease in fat mass with high dosage probiotics (-1.08 [-1.21, -0.95] kg) compared to low dosage probiotics (-1.00 [-1.59, -0.42] kg), a greater decrease with single strain probiotics (-1.15 [-1.28, -1.02] kg) compared to multiple strain probiotics (-0.60 [-0.94, -0.26] kg), and a greater decrease with administration probiotics in the form of food (-1.13 [-1.58, -0.67] kg) compared to in the forms of capsule or powder (-1.07 [-1.20, -0.94] kg). Due to the fact that few trials reported the effect of probiotics on fat percentage, subgroup analyses were not performed to investigate the source of heterogeneity. No particular study significantly affected the pooled effect of probiotics on fat mass and fat percentage by sensitivity analyses.

There was no sign of publication bias on the effect of probiotics to fat mass (Egger's test* P* = 0.335) and fat percentage (Egger's test* P* = 0.068). The funnel plots were presented in Figure S1.

#### 3.3.2. Probiotics and Lipid Profiles (TC, TG, LDL-C, and HDL-C Level)

**Figure 4:**
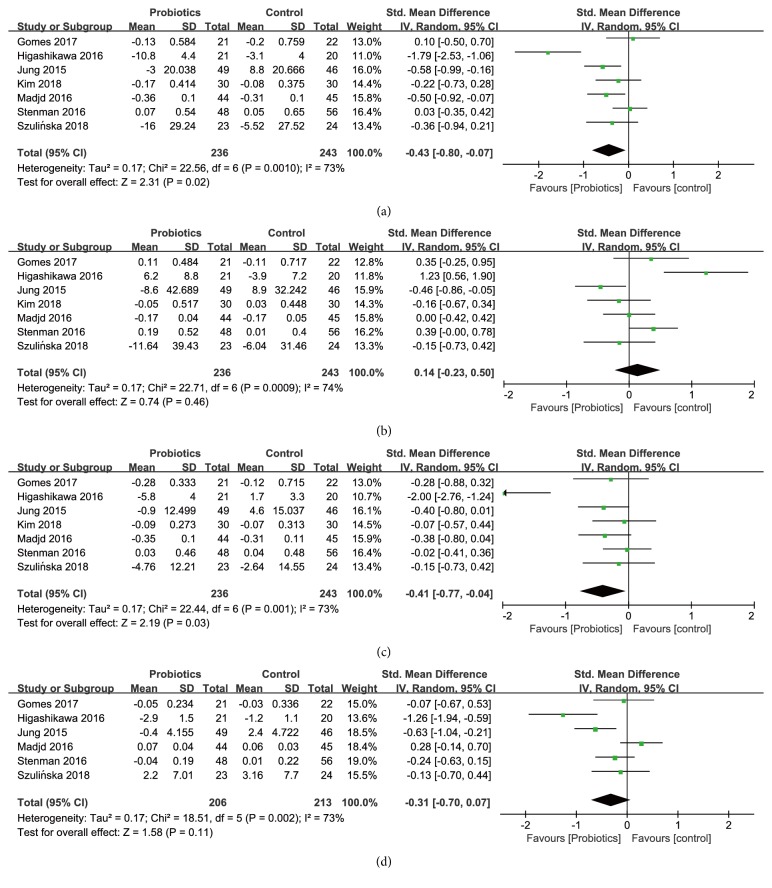
Forest plot of effect of probiotics on (a) TC; (b) TG; (c) LDL-C; (d) HDL-C.

*Effects on Total Cholesterol (TC)*. Seven studies, among 479 participants (236 consuming probiotics, 243 not consuming probiotics), reported the effect of probiotics supplementation on TC (Figure 4(a)). There was a statistically significant reduction in the intervention group with a pooled standardized mean difference of -0.43 (SMD [95%* CI*]; [-0.80, -0.07],* P* = 0.02) with a moderate heterogeneity (*I*^*2*^ = 73%,* P* = 0.001) between the studies.

Because there were no studies that reported the effect of probiotics on TC in the form of food, subgroup analyses (Table 6) only stratified by probiotics dosage and the number of probiotics strains indicated the effects of probiotics supplementation on TC were significantly reduced in trials with single strain probiotics (-0.61 [-1.54, -0.32]), compared to multiple strain probiotics (-0.39 [-0.66, -0.13]). Sensitivity analyses revealed that no particular studies significantly affected the summary effects of TC.

There was no sign of publication bias analyzed by Egger's test for the effect of probiotics on TC (*P* = 0.276), and the funnel plot was presented in Figure S2. 

*Effects on Triglyceride (TG)*. A total of 7 studies including 479 participants (236 consuming probiotics, 243 not consuming probiotics) evaluated the effect of probiotics supplementation on TG. There was no statistically significant reduction in TG levels (Figure 4(b)) with a pooled standardized mean difference of 0.14 (SMD [95% CI]; [-0.23, 0.50], P=0.46) and a moderate heterogeneity (*I*^*2*^=74%, P=0.0009).

Subgroup analyses (Table 7) stratified by probiotics dosage and the number of probiotics strains indicated the effects of probiotics supplementation on TG were not significantly reduced in subgroup analyses. Sensitivity analyses revealed that no particular studies significantly affected the summary effects of TG.

There was no sign of publication bias of the effect of probiotics on TG (Egger's test* P* = 0.300), and the funnel plot was presented in Figure S2. 

*Effects on Low Density Lipoprotein Cholesterol (LDL-C*). Seven studies among 479 participants (236 consuming probiotics, 243 not consuming probiotics) reported the changes of LDL-C (Figure 4(c)). Comparing with the control group the pooled standardized mean difference showed a statistically significant reduction in LDL-C in probiotics group (SMD [95%* CI*]; -0.41 [-0.77, -0.04],* P* = 0.03) with a moderate heterogeneity (*I*^*2*^ = 73%,* P* = 0.001).

Subgroup analyses (Table 8) stratified by probiotics dosage and the number of probiotics strains indicated the effects of probiotics supplementation on LDL-C were significantly reduced in trials with multiple strain probiotics (-0.33 [-0.57, -0.09]). Sensitivity analyses revealed that no particular studies significantly affected the summary effects of LDL-C.

There was no sign of publication bias of the effect of probiotics on LDL-C (Egger's test* P* = 0.124), and the funnel plot was presented in Figure S2. 

*Effects on High Density Lipoprotein Cholesterol (HDL-C)*. A total of 6 studies including 419 participants (206 consuming probiotics, 213 not consuming probiotics) evaluated the effect of probiotics supplementation on HDL-C. Effect of probiotics on TG levels showed weak significance (Figure 4(d)) with a pooled standardized mean difference of -0.31 (SMD [95%* CI*]; [-0.70, 0.07],* P*=0.11) and a moderate level of heterogeneity (*I*^*2*^=73%,* P*=0.002). Due to no more than 7 studies reported on the effects of probiotics on HDL-C, subgroup analyses were not conducted. No particular study significantly affected the pooled effect of probiotics on HDL-C by sensitivity analyses.

There was no sign of publication bias analyzed by Egger's test for the effect of probiotics on HDL-C (*P* = 0.484).

#### 3.3.3. Probiotics and Glucose Metabolism (Fasting Plasma Glucose, HbA1c, and HOMA-IR)

**Figure 5:**
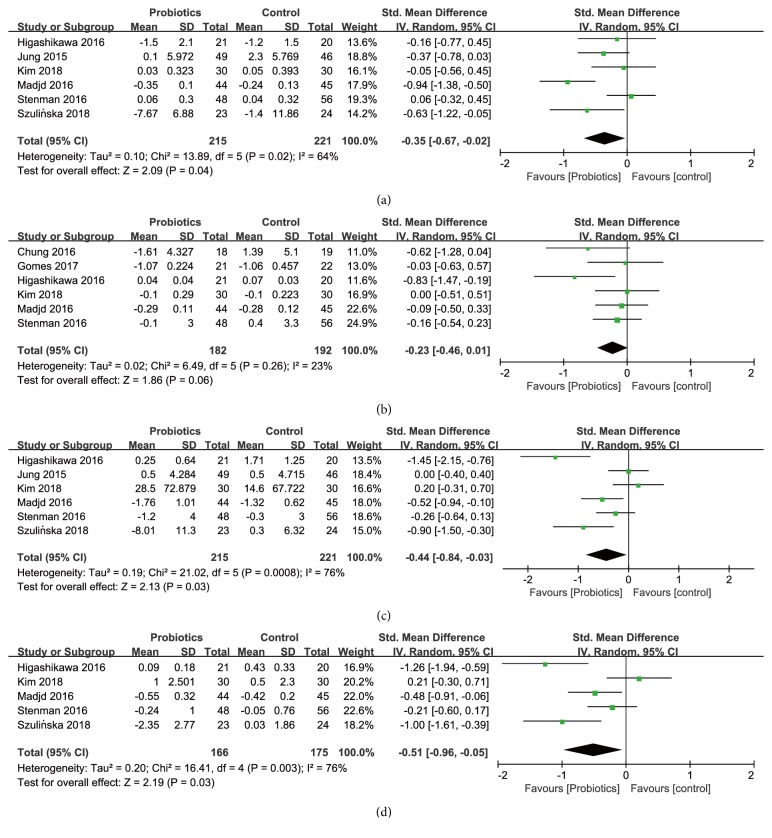
Forest plot of effect of probiotics on (a) fasting plasma glucose; (b) HbA1c; (c) insulin; (d) HOMA-IR.

*Effects on Fasting Plasma Glucose (FPG*). Six studies among 436 participants (215 consuming probiotics, 221 not consuming probiotics) reported on changes in fasting plasma glucose (Figure 5(a)), and the pooled standardized mean difference showed a statistically significant reduction in fasting plasma glucose in probiotics group (SMD [95%* CI*]; -0.35 [-0.67, -0.02],* P* = 0.04) compared with the control group, with a moderate heterogeneity (*I*^*2*^ = 64%,* P* = 0.02).

There was no sign of publication bias analyzed by Egger's test for the effect of probiotics on FPG (Egger's test* P* = 0.791). 

*Effects on Hemoglobin A1c (HbA1c)*. Six studies including 374 participants (182 consuming probiotics, 192 not consuming probiotics) reported on changes in HbA1c (Figure 5(b)), and the pooled standardized mean difference showed a weak trend toward significance in HbA1c in probiotics group (SMD [95%* CI*]; -0.23 [-0.46, 0.01],* P* = 0.06) compared with the control group, with a low level of heterogeneity (*I*^*2*^ = 23%,* P* = 0.26).

There was no sign of publication bias analyzed by Egger's test for the effect of probiotics on HbA1c (Egger's test* P* = 0.190). 

*Effects on Insulin*. A total of 6 studies including 436 participants (215 consuming probiotics, 221 not consuming probiotics) evaluated the effect of probiotics supplementation on insulin (Figure 5(c)). In comparison with the control group after treatment, the reduction of insulin was statistically significant with a pooled standardized mean difference of -0.44 (SMD [95%* CI*]; [-0.84, -0.03],* P* = 0.03) in the probiotics group, with a high level of heterogeneity (*I*^*2*^ = 76%,* P* = 0.0008) between the studies.

There was no sign of publication bias analyzed by Egger's test for the effect of probiotics on insulin (Egger's test* P* = 0.133). 

*Effects on Homeostasis Model of Assessment for Insulin Resistance Index (HOMA-IR)*. A total of 5 studies, with 341 participants (166 consuming probiotics, 175 not consuming probiotics), evaluated the effect of probiotics supplementation on HOMA-IR (Figure 5(d)). Effect of probiotics on HOMA-IR level was shown to be statistically significant, with a pooled standardized mean difference of -0.51 (SMD [95%* CI*]; [-0.96, -0.05],* P*=0.03) and a high level of heterogeneity (*I*^*2*^=76%,* P*=0.003).

There was no sign of publication bias analyzed by Egger's test for the effect of probiotics on HOMA-IR (Egger's test* P* = 0.244). 

*Subgroup Analyses and Sensitivity Analyses*. Due to no more than 7 studies reported on the effects of probiotics on fasting plasma glucose, HbAlc, insulin, and HOMA-IR, subgroup analyses were not conducted based on probiotics dosage, numbers of probiotics species, and forms of probiotics. No particular study significantly affected the pooled effect of probiotics on glucose metabolic parameters (fasting plasma glucose, HbA1c, and HOMA-IR) by sensitivity analyses.

## 4. Discussion

### 4.1. Summary of Our Study

In this study, we performed a systematic review and meta-analysis to examine the effects of probiotics supplementation in healthy adults with overweight/obesity. The results suggest that probiotics have positive effects on weight-loss in parallel with the improvement of the plasma lipid profile and glucose metabolism.

In regard to probiotics and weight-loss, 12 randomized controlled trials were included in the meta-analysis, of which 10 studies reported the changes in body weight, 11 studies reported the changes in BMI, 8 studies reported the changes in waist circumference, 9 studies reported the changes in fat mass, and 6 studies reported the changes in fat percentage. As expected, the body weight reduction was significant in probiotics group with a pooled mean difference (WMD [95%* CI*]; -0.55 [-0.91, -0.19] kg), BMI (WMD [95%* CI*]; -0.30 [-0.43, -0.18] kg m^−2^), waist circumference (WMD [95%* CI*]; -1.20 [-2.21, -0.19] cm), fat mass (WMD [95%* CI*]; -0.91 [-1.19, -0.63] kg), and fat percentage (WMD [95%* CI*]; -0.92 [-1.27, -0.56] %). Different from studies on patients with diabetes or other metabolism syndromes [27], there was also a significant reduction in fat mass and fat percentage in healthy adults.

To characterize the effects of probiotics on plasma lipid profiles, 7 randomized controlled trials were included in the meta-analysis, of which 7 studies reported the changes in TC, TG, or LDL-C, respectively, and 6 studies reported the changes in HDL-C. Similar to the previous study on patients with diabetes or other metabolism syndromes [23, 33], probiotics supplementation also significantly reduced TC (SMD [95%* CI*]; -0.43 [-0.80, -0.07]) and LDL-C (SMD [95%* CI*]; -0.41 [-0.77, -0.04]); however, the changes in TG (SMD [95%* CI*]; 0.14 [-0.23, 0.50]) and HDL-C (SMD [95%* CI*]; -0.31 [-0.70, 0.07]) were not significant.

Regarding the changes in glucose metabolism, 8 randomized controlled trials were included in the meta-analysis, of which 6 studies reported the changes in PFG, HbA1c, and insulin, respectively. 5 studies reported changes in HOMA-IR. Our findings support the probiotics supplementation could improve the glucose metabolism, which was similar to previous reports in patients with diabetes [33, 34]. Statistically significant reduction was found on FPG (SMD [95%* CI*]; -0.35 [-0.67, -0.02]), insulin (SMD [95%* CI*]; -0.44 [-0.84, -0.03]), and HOMA-IR (SMD [95%* CI*]; -0.51 [-0.96, -0.05]), respectively. However, there was no statistically significant reduction in HbA1c levels with a pooled standardized mean difference of HbA1c (SMD [95%* CI*]; -0.23 [-0.46, 0.01]).

### 4.2. The Possible Mechanism

One of the potential mechanisms that has been proposed to explain the results is that through the gut microbiota altered by the probiotics [3]. The probiotics supplementation might increase the short-chain fatty acids (SCFAs) producing bacteria, decrease the abundance of lipopolysaccharide (LPS) producers, and relieve the tissue and organic inflammation induced by LPS [3, 8, 35, 36]. The probiotics might also reduce the opportunistic pathogens and their metabolites, such as trimethylamine (TMA), LPS, and indole [37]. Probiotics also could reduce the fat accumulation, downregulate inflammation levels, and improve the insulin sensitivity accompanied by the increase of the neuropeptides and gastrointestinal peptides and the abundance of several beneficial bacteria [4, 8, 9, 38, 39]. As a result, metabolic homeostasis would be improved to keep healthy.

### 4.3. Strengths and Limitations

To the best of our knowledge, no previous study has systematically reviewed and analyzed the effects of probiotics on weight-loss in healthy adults with a BMI > 25 kg m^−2^. This study firstly assessed the effects of probiotics on obesity and the associated clinical indicators, such as plasma lipid profiles and glycaemic parameters. In order to get an accurate result, the studies in which participants consumed prebiotics, synbiotics, herb, and other supplements (such as micronutrients or other dietary constituents) were excluded as the effects of probiotics supplementation from those supplementations could not be distinguished; also the possible interaction between probiotics supplementation and those supplementations was excluded.

There are also some limitations in this study. The majority of the included trials were of small size, and four clinical trials were not registered in a clinical trial registry, which may have a risk of reporting bias. One study did not report the species and dose of probiotics supplementation. Besides, the survivability and stability of probiotics, influenced by the manufacturers, could also affect the clinical outcomes. Due to the limited number of included trials, the effects of probiotics supplementation in the prevention and treatment of overweight/obesity still need more intensive work.

## 5. Conclusions

In summary, our work suggests that probiotics supplementation could reduce the body weight and fat mass and improve some of the lipid and glucose metabolism parameters, although some of the effects were small. Probiotics may become a new potential strategy for the prevention and treatment of overweight/obesity in adult individuals.

## Figures and Tables

**Table 1:** Characteristics of the included studies.

Studies	Country	No. of participants (P/C)	Trial Number	ITT	Study design	Form of probiotics	Probiotics strains	Daily dose (CFU)	Duration (Weeks)
Szulińska 2018[31]	Poland	47 (24/23)	NCT03100162	No	DB, PC	Sachet	Multi (*Bifidobacterium bifidum* W23, *Bifidobacterium lactis* W51, *Bifidobacterium lactis* W52,* Lactobacillus acidophilus* W37, *Lactobacillus brevis* W63, *Lactobacillus casei* W56, *Lactobacillus salivarius* W24, *Lactococcus lactis* W19, and *Lactococcus lactis* W58)	1×10^10^	12

Kim 2018[13]	Korea	60 (30/30)	KCT0000756	Yes	DB, PC	Capsule	Single (*Lactobacillus gasseri* BNR17)	1×10^10^	12

Gomes 2017[14]	Brazil	43 (22/21)	U1111-1137-4566	No	DB, PC	Sachet	Multi (*Lactobacillus acidophilus* LA - 14, *Lactobacillus casei* LC - 11, *Lactococcus lactis* LL - 23, *Bifidobacterium bifidum* BB - 06, and *Bifidobacterium lactis* BL - 4)	2×10^10^	8

Kim 2017[15]	Korea	66 (34/32)	NCT02492698	No	DB, PC	Powder	Multi (*Lactobacillus curvatus* HY7601 and *Lactobacillus plantarum* KY1032)	5×10^9^	12

Stenman 2016[20]	Finland	104 (56/48)	NCT01978691	ITT	DB, PC	Sachet	Single (*Bifidobacterium animalis ssp. lactis* 420)	1×10^10^	26*∗*

Higashikawa 2016[32]	Japan	41 (20/21)	/	ITT	DB, PC	Powder	Single (*Pediococcus pentosaceus* LP28)	1×10^11^	12

Chung 2016[16]	Korea	37 (19/18)	KCT0000452	No	DB, PC	Capsule	Single (*Lactobacillus* JBD301)	1×10^9^	12

Fathi 2016[21]	Iran	50 (25/25)	IRCT2013061313661N1	ITT	DB, PC	Kefir	/	/	8

Madjd 2016[22]	Iran	89 (45/44)	IRCT201402177754N8	ITT	SB, PC	Yogurt	Multi (*Lactobacillus acidophilus* LA5, *Bifidobacterium lactis* BB12)	1×10^7^	12

Jung 2015[17]	Korea	95 (46/49)	/	No	DB, PC	Powder	Multi (*Lactobacillus curvatus* HY7601 and *Lactobacillus plantarum* KY1032)	1×10^10^	12

Zarrati 2014[18]	Iran	50 (25/25)	/	ITT	DB, PC	Yogurt	Multi (*Lactobacillus acidophilus* La5, *Bifidobacterium* BB12, and *Lactobacillus casei* DN001)	6×10^9^	8

Kadooka 2013[19]	Japan	139 (70/69)	/	ITT	DB, PC	Fermented milk	Single (*Lactobacillus gasseri* SBT2055)	2×10^11^	12

P/C, probiotic group/control group; ITT/PP, Intention-To-Treat/Per-Protocol; DB, double blinded; PC, placebo controlled; SB, single blinded; CFU, colony-forming units; *∗* 6 months.

**Table 2:** Subgroup analyses for the effects of probiotics on body weight.

Subgroup	Number of trials	Number of participants	*I* ^*2*^	Weighted mean difference
			%	(95% *CI*)
Probiotics dosage				
≥ 10^10^ CFU	5	171	3.8	-0.58 (-0.92, -0.23)
< 10^10^ CFU	4	119	75.0	-0.41(-1.15, 0.34)
Number of probiotics species				
Single	3	96	0	-0.49 (-0.92, -0.07)
Multiple	6	194	68.5	-0.41 (-0.97, 0.14)
Form of probiotics				
Capsule or powder	7	247	19.4	-0.55(-0.84, -0.26)
Food	3	68	87.5	-0.50(-1.68, 0.67)

**Table 3:** Subgroup analyses for the effects of probiotics on BMI.

Subgroup	Number of trials	Number of participants	*I* ^*2*^	Weighted mean difference
			%	(95% *CI*)
Probiotics dosage				
≥ 10^10^ CFU	6	213	66.6	-0.29 (-0.46, -0.12)
< 10^10^ CFU	4	119	46.9	-0.18 (-0.48, 0.12)
Number of probiotics species				
Single	4	138	56.8	-0.36 (-0.52, -0.20)
Multiple	6	194	55.5	-0.15 (-0.39, 0.10)
Form of probiotics				
Capsule or powder	7	220	64.1	-0.25(-0.43, -0.07)
Food	4	137	61.4	-0.34(-0.57, -0.12)

**Table 4:** Subgroup analyses for the effects of probiotics on waist circumference.

Subgroup	Number of trials	Number of participants	*I* ^*2*^	Weighted mean difference
			%	(95% *CI*)
Probiotics dosage				
≥ 10^10^ CFU	6	212	86.9	-1.53 (-2.64, -0.41)
< 10^10^ CFU	2	69	0	-0.32 (-0.84, 0.20)
Number of probiotics species				
Single	4	168	91.6	-1.69 (-3.04, -0.33)
Multiple	4	113	0	-0.42 (-0.91, 0.07)
Form of probiotics				
Capsule or powder	6	187	92.3	-1.34 (-2.76, 0.08)
Food	2	94	0	-1.11 (-1.64, -0.59)

**Table 5:** Subgroup analyses for the effects of probiotics on fat mass.

Subgroup	Number of trials	Number of participants	*I* ^*2*^	Weighted mean difference
			%	(95% *CI*)
Probiotics dosage				
≥ 10^10^ CFU	7	261	52.5	-1.08 [-1.21, -0.95]
< 10^10^ CFU	2	50	27.2	-1.00 [-1.59, -0.42]
Number of probiotics species				
Single	5	186	0	-1.15 [-1.28, -1.02]
Multiple	3	125	0	-0.60 [-0.94, -0.26]
Form of probiotics				
Capsule or powder	6	224	53.0	-1.07 [-1.20, -0.94]
Food	2	87	20.0	-1.13 [-1.58, -0.67]

**Table 6:** Subgroup analyses for the effects of probiotics on TC.

Subgroup	Number of trials	Number of participants	*I* ^*2*^	Standardized mean difference
			%	(95% *CI*)
Probiotics dosage				
≥ 10^10^ CFU	6	192	77.4	-0.43 [-0.87, 0.01]
< 10^10^ CFU	1	44	/	-0.50 [-0.92, -0.07]
Number of probiotics species				
Single	3	99	89.3	-0.61 [-1.54, -0.32]
Multiple	4	137	16.8	-0.39 [-0.66, -0.13]

**Table 7:** Subgroup analyses for the effects of probiotics on TG.

Subgroup	Number of trials	Number of participants	*I* ^*2*^	Standardized mean difference
			%	(95% *CI*)
Probiotics dosage				
≥ 10^10^ CFU	6	192	77.8	0.17 [-0.27, 0.61]
< 10^10^ CFU	1	44	/	0.00 [-0.42, -0.42]
Number of probiotics species				
Single	3	99	81.0	0.45 [-0.23, 1.13]
Multiple	4	137	43.4	-0.10 [-0.43, 0.22]

**Table 8:** Subgroup analyses for the effects of probiotics on LDL-C.

Subgroup	Number of trials	Number of participants	*I* ^*2*^	Standardized mean difference
			%	(95% *CI*)
Probiotics dosage				
≥ 10^10^ CFU	6	192	77.6	-0.43 [-0.87, 0.02]
< 10^10^ CFU	1	44	/	-0.38 [-0.80, 0.04]
Number of probiotics species				
Single	3	99	90.9	-0. 64 [-1.66, 0.37]
Multiple	4	137	73.3	-0.33 [-0.57, -0.09]

## Abbreviations

BMI: Body mass index
WMD: Weighted mean difference
SMD: Standardized mean difference
TC: Total cholesterol
TG: Triglyceride
HDL-C: High density lipoprotein cholesterol
LDL-C: Low density lipoprotein cholesterol
FPG: Fasting plasma glucose
HbA1c: Hemoglobin A1c
HOMA-IR: Homeostasis model of assessment for insulin resistance index.

## Supplementary Materials

Supplementary 1 Table S1: the search strategy.

Supplementary 2 Figure S1: the funnel plots of effect of probiotics on (A) body weight; (B) BMI; (C) BMI modified by the “trim and fill” method; (D) waist circumference; (E) fat mass; (F) fat percentage.

Supplementary 3 Figure S2: the funnel plots of effect of probiotics on (A) total cholesterol; (B) triglyceride; (C) lipoprotein cholesterol.
