# Supplementary material for: The Potential Role of Probiotics in Controlling Overweight/Obesity and Associated Metabolic Parameters in Adults: A Systematic Review and Meta-Analysis
Source: Evid Based Complement Alternat Med. 2019 Apr 15;2019:3862971. doi: 10.1155/2019/3862971 (PMC6500612; doi:10.1155/2019/3862971)
Supplement: Supplementary 1 — Table S1: the search strategy. [file 3862971.f1.doc]

**Table S1.** Search terms

| **PubMed (Medline)** |
| --- |
| Search hits 191  Search (“Probiotics”[Mesh] OR probiotic*[tw] OR “Synbiotics”[Mesh] OR synbiotic*[tw] OR “Lactobacillus”[Mesh] OR lactobacillus[tw] OR “Bifidobacterium”[Mesh] OR bifidobacterium[tw] OR bifidobacteria[tw]) AND (“Body Weight Changes”[Mesh] OR weight change*[tw] OR weight gain*[tw] OR weight loss*[tw] OR weight regulation*[tw] OR “weight modification”[tw] OR “Body Weight Maintenances”[Mesh] or Weight Maintenance*[tw] OR “Obesity”[Mesh] or obes*[tw] OR “Obesity Managements”[Mesh] OR “Obesity Management”[tw] OR “Overweight”[Mesh] OR overweight[tw])  Filters: Clinical Trial; published in the last 10 years |
| **Embase** |
| Search hits 719 #3 #1 AND #2  #1 AND ('clinical article'/de OR 'clinical trial'/de OR 'controlled clinical trial'/de OR 'major clinical study'/de OR 'randomized controlled trial'/de OR 'randomized controlled trial (topic)'/de) AND (2008:py OR 2009:py OR 2010:py OR 2011:py OR 2012:py OR 2013:py OR 2014:py OR 2015:py OR 2016:py OR 2017:py OR 2018:py)  #2 ('probiotic agent'/exp OR probiotic*:ti,ab OR 'prebiotic agent'/exp OR prebiotic*:ti,ab OR 'synbiotic agent'/exp OR synbiotic*:ti,ab OR 'lactobacillus'/exp OR lactobacillus:ti,ab OR 'bifidobacterium'/exp OR 'bifidobacterium':ti,ab OR 'bifidobacteria':ti,ab) AND ('weight change'/exp OR 'weight change*':ti,ab OR 'weight gain*':ti,ab OR 'weight loss*':ti,ab OR 'weight regulation*':ti,ab OR 'weight modification':ti,ab OR 'weight maintenance*':ti,ab OR 'obesity'/exp OR obes*:ti,ab OR 'obesity management':ti,ab OR overweight:ti,ab) |
| **Web of Science** |
| Search hits 338  TS = (probiotic* OR synbiotic* OR lactobacillus OR bifidobacterium OR bifidobacteria) AND TS = (weight change* OR weight gain* OR weight loss* OR weight regulation* OR weight modification OR weight maintenances OR obes* OR overweight)  Refined by: Document Types: ( CLINICAL TRIAL ) Timespan: 2008-2018 |
